# Enhancing the diagnosis and management of COPD in Primary care

**DOI:** 10.1186/2049-6958-9-62

**Published:** 2014-11-28

**Authors:** Martyn R Partridge

**Affiliations:** Airway Diseases Section, National Heart and Lung Institute, Imperial College London, Dovehouse Street, London, SW3 6LY UK

Achieving the optimal management of Chronic Obstructive Pulmonary Disease (COPD) is a major challenge facing all countries where smoking is and has been highly prevalent. The consequences of missed or delayed diagnosis represent a source of distress to patients, unnecessary expense to health care systems, and a missed opportunity to implement the many things we can now do to reduce the burden of this disease. Others have described the story of COPD as being a story with no beginning, a middle that is a way of life, and an unpredictable and unanticipated end [1]. If we are to give the story a beginning it can be achieved either by the recognition of symptoms leading to an accurate objective diagnosis, or less satisfactorily by a major event such as an exacerbation starting the story. In the latter case the opportunity for support with smoking cessation, pulmonary rehabilitation and initiation of guideline recommended therapies is missed. The immense value of flu vaccination, stop smoking support, pulmonary rehabilitation and simple bronchodilator therapy, compared to more complex therapies and interventions, has been graphically displayed in the P*yramid of value for COPD interventions* to which further attention has been drawn recently [2]. So how do we start the story earlier to ensure that those with COPD are offered and take these cost effective interventions?

In the article recently published in *Multidisciplinary Respiratory Medicine* [3] Sanguinetti and colleagues [3] report a nationwide study of this and related issues and whilst highlighting an appreciation of the role of spirometry in specialist and General Practice they report on the latters concern re difficulty in accessing spirometry and other barriers to implementation. This by itself is not new and others have demonstrated how easier access to well conducted spirometry can reveal how clinical impressions are insufficient to make a diagnosis and mistakenly lead to those with no evidence of airway narrowing receiving often expensive bronchodilators [4]. It is thus a challenge for all countries to address how well conducted spirometry can be promptly available and Sanguinetti’s study is encouraging in that it demonstrates GP recognition of this need. Others have also demonstrated GPs awareness of this, and that of Junior Doctors, although the latter report losing interest if specialists do not pay attention to the results [5]. It is quite possible for good quality spirometry to be undertaken safely and interpreted correctly in primary care but in the same way that arrangements for provision of Chest Radiographs are organised by locality, conducted to a high standard and reported accurately, a similar more central service for spirometry, if promptly available, would clearly be appreciated by General Practitioners. This is not however the end of the story. Case finding is essential if patients are to be referred for spirometry and accurate recording of smoking habits during all consultations for whatever cause, provides a data base of those needing to be investigated. In those presenting with suggestive symptoms a second caveat is to be aware of the problem of the patient with significant breathlessness who has only mild obstruction and where the brunt of damage is borne by the alveoli — dominant air sac disease or emphysema. Recording a breathlessness score at the time of performing spirometry can permit the reporter to highlight this possibility to the primary care physician, and some of these patients clearly need more detailed lung function tests and CT scans. Obesity as a confounding cause of breathlessness is also underappreciated [4].

In the ideal world a prompt diagnosis is made, the patient supported to stop smoking, appropriate vaccinations given and the patient referred for pulmonary rehabilitation to a programme that appreciates the importance of motivating the patient and supporting them as they self manage their own condition following appropriate behavioural change. Not all will require pharmacological interventions other than smoking cessation treatments, but many who are symptomatic will require a minimum of bronchodilator therapy. As Sanguinetti points out this and other regular therapies then need to be monitored by prescription monitoring, for non-adherence is common in long term conditions [6] and more focus on optimal doctor patient communication, determining the patients goals, shared decision making and permitting the patient to ask about any concerns regarding side effects is essential. Even within a large clinical trial when those with COPD knew that their use of therapy was being monitored a fifth of patients did not take medicine as previously discussed with the doctor with one trial showing a significantly deleterious effect of this behaviour on hospitalisation rates and upon mortality [7]. The Pneumocafé project [3] represents a further way of demonstrating the importance of this subject to primary care physicians.

Integrated care approaches to the management of this common disease are proven to improve outcomes [8] and in a number of formats appropriate to a country’s health care system must surely be the way forward?
